# Targeted therapy based on ubiquitin-specific proteases, signalling pathways and E3 ligases in non-small-cell lung cancer

**DOI:** 10.3389/fonc.2023.1120828

**Published:** 2023-03-09

**Authors:** Yu-Chen Yang, Can-Jun Zhao, Zhao-Feng Jin, Jin Zheng, Li-Tian Ma

**Affiliations:** ^1^ Department of Traditional Chinese Medicine, Tangdu Hospital, Air Force Medical University, Xi’an, China; ^2^ School of Psychology, Weifang Medical University, Weifang, China; ^3^ Department of Gastroenterology, Tangdu Hospital, Air Force Medical University, Xi’an, China

**Keywords:** NSCLC, USPs, PROTACs, E3 ligase, signalling pathway

## Abstract

Lung cancer is one of the most common malignant tumours worldwide, with the highest mortality rate. Approximately 1.6 million deaths owing to lung cancer are reported annually; of which, 85% of deaths occur owing to non-small-cell lung cancer (NSCLC). At present, the conventional treatment methods for NSCLC include radiotherapy, chemotherapy, targeted therapy and surgery. However, drug resistance and tumour invasion or metastasis often lead to treatment failure. The ubiquitin–proteasome pathway (UPP) plays an important role in the occurrence and development of tumours. Upregulation or inhibition of proteins or enzymes involved in UPP can promote or inhibit the occurrence and development of tumours, respectively. As regulators of UPP, ubiquitin-specific proteases (USPs) primarily inhibit the degradation of target proteins by proteasomes through deubiquitination and hence play a carcinogenic or anticancer role. This review focuses on the role of USPs in the occurrence and development of NSCLC and the potential of corresponding targeted drugs, PROTACs and small-molecule inhibitors in the treatment of NSCLC.

## 1 Introduction

Lung cancer is one of the most common primary malignant tumours. Non-small-cell lung cancer (NSCLC) refers to any type of epithelial lung cancer except for small cell lung cancer. It accounts for approximately 85% of the total lung cancer cases. NSCLC is primarily divided into two types: lung squamous cell carcinoma (LUSC) and lung adenocarcinoma (LUAD) (1). Recent studies have demonstrated that uncontrolled intracellular protein homeostasis, especially abnormality of the ubiquitin–proteasome pathway (UPP), plays an important role in the progression of NSCLC. Deubiquitinating enzymes (DUBs) play an important role in UPP, which is responsible for removing the ubiquitin chain of protein substrate in cells. Abnormal activity or expression of DUBs can cause functional changes in key carcinogenic/tumour suppressor proteins, leading to malignant transformation of cells (2, 3). Ubiquitin-specific proteases (USPs), the largest subset of the DUBs family, are expressed in different cancer cells and mediate deubiquitination (4). Various USPs are abnormally expressed in NSCLC cells, with most USPs having evident carcinogenic effects and only a few USPs having beneficial effects (5–9). Targeting USPs with carcinogenic effects may benefit patients with NSCLC. Furthermore, E3 ligases mediate the binding of target proteins to the proteasome, and enhancing or inhibiting their activity can affect the degradation of target proteins. Therefore, E3 ligases may participate in regulating the activity of tumour cells (10). In this review, we summarised the role and mechanisms of action of USPs in NSCLC, the effects of enhancing/reducing the activity of target proteins through UPP and the potential of small-molecule inhibitors and PROTACs technology in the treatment of NSCLC.

## 2 Ubiquitination and deubiquitination

Eukaryotic cells recognize and degrade proteins through UPP. Substrate proteins bound to ubiquitin chains are directed to the 26S proteasome for degradation. UPP is a major protein breakdown mechanism in mammalian cells. The 26S proteasome consists of a 19S regulatory complex, which is responsible for recognizing ubiquitinated proteins, and a 20S protein breakdown core, which is primarily responsible for catalysing protein degradation (11). Ubiquitination can be classified as monoubiquitination, in which a Ub molecule is directly added to the lysine residue of a protein, and polyubiquitination, in which a Ub chain is formed from a single lysine residue on a substrate. The lysine residue and the N-terminal methionine residue (M1) of Ub can serve as ubiquitination sites and bind to Ub molecules to form different types of Ub chains. Seven lysine residues, namely, K6, K11, K27, K29, K33, K48 and K63, are known to be amenable to ubiquitination. K48- and K11-linked polyubiquitin chains are mainly related to proteasomal degradation, whereas K63-linked polyubiquitin chains are mainly involved in signal transduction in cells (12, 13). E1 enzymes initiate the modification of ubiquitination by adenylating Ub using ATP to form a high-energy thioester bond between the C-terminal carboxyl group of Ub and the thiol group of the cysteine residue of E1. The activated Ub is subsequently transferred to the cysteine residue of an E2 enzyme to form a similar thioester bond. Finally, an E3 ligase recruits the E2 enzyme to facilitate the specific transfer of Ub to substrate proteins, resulting in degradation of target proteins by the proteasome (14). Ubiquitination is involved in several biological processes, including but not limited to: enzyme activation, regulatory interaction between proteins, signal transduction and regulation of transcription (15).

E3 ligases are the key enzymes in ubiquitination, which determine the reaction rate and substrate properties (16). E3 ligases found in mammalian cells are categorised into three types according to their structural characteristics and mechanisms of action: really interesting new genes (RINGs), homologous to E6AP C-terminus (HECT) and RING-between-RING (RBR) ligases (17). RING E3 ligases catalyse the direct transfer of Ub from E2 enzymes to the substrate, whereas HECT and RBR E3 ligases contain a catalytic form of cysteine that receives Ub from E2 enzymes and transfers it to the target protein. Studies have demonstrated that E3 ligases are closely related to tumour development and may be involved in tumour invasion, cell proliferation, apoptosis, DNA damage and repair, tumour metabolism and immunity (10). The effects of E3 ligases vary based on the target protein they bind to. For example, Pirh2, the product of the human *RCHY1* gene, is an E3 ligase containing a ring structure that stabilises *c-MYC* and promotes the growth, invasive ability and migratory ability of NSCLC (H1299) cells (18). Homologous to the E6-associated protein carboxyl terminus domain-containing 3 (HECTD3) is a member of the HECT E3 ligase family. Its overexpression primarily regulates K63 polyubiquitination and promotes MALT1 stabilisation, which promotes the proliferation of angiotensin II receptor-positive breast cancer cells (19, 20). Therefore, E3 ligases may play an oncogenic role. Knockdown of the E3 ligase MKRN3 can increase the proliferation of NSCLC cells, whereas upregulation of recombinant MKRN3 can directly inhibit the growth and proliferation of NSCLC cells both *in vitro* and *in vivo*. Therefore, E3 ligases may mediate the action of MKRN3 and have certain tumour-suppressing effects (21). E3 ligases mediate the degradation of substrate proteins and exert both protective and detrimental effects. However, whether the degradation of substrate proteins mediated by E3 ligases promotes or inhibits tumours remains unclear.

Ubiquitination and deubiquitination are two mechanisms for modifying protein homeostasis in cells. These mechanisms have controllable and reciprocal characteristics and cooperate to dynamically regulate various cellular processes (22). DUBs catalyse deubiquitination and disrupt the bond between Ub molecules and substrate proteins, thus acting as proofreaders for protein degradation and preventing abnormal hydrolysis of active proteins. The human genome encodes approximately 100 DUBs, which can be roughly divided into two categories based on their catalytic domain: cysteine proteases and metalloproteinases. The seven subfamilies of DUBs are as follows: USPs, ubiquitin carboxyl-terminal hydrolases (UCHs), ovarian tumour domain proteases (OTUs), Machado–Joseph disease protein domain-containing proteases (MJDs/Josephins), motif− interacting with ubiquitin−containing novel DUB family (MINDYs) and zinc finger-containing ubiquitin peptidase1 (ZUP1) belong to cysteine proteases, whereas JAB1/MPN/Mov34 metalloenzymes (JAMMs) belong to metalloproteinases (23–25). The deubiquitination mechanism of cysteine proteases depends on the conserved catalytic triad of amino acids, His-Cys-Asn/Asp. Histidine (His) residues reduce the pKa of cysteine (Cys) residues and promote the nucleophilic attack of Cys residues on isopeptide bonds, whereas Asn/Asp residues usually play a role in the localisation and polarisation of His residues (26). The deubiquitination mechanism of metalloproteinases depends on the coordination of His, aspartic acid and serine residues with zinc ions (27).

USPs are the largest subset of the DUBs family, and approximately 70 USPs have been identified in humans. The functions of USPs are similar to those of DUBs. USPs can recognise various substrates with Lys48-, Lys63- and Met1-linked Ub chains, which are involved in several processes related to tumour progression, including epithelial–mesenchymal transition (EMT), tumour metastasis, alterations in the tumour microenvironment, DNA damage repair and protein dysfunction (4, 28, 29). USPs possess a highly conserved USP domain consisting of three subdomains that resemble the palm, thumb and finger of the right hand, respectively. The catalytic site of USPs is located between the palm and thumb regions, and the catalytic centre of USPs includes a C-terminal His Box and an N-terminal Cys Box with catalytic His and Cys residues, respectively, at the interface between the thumb and palm subdomains. The finger region is responsible for interaction with Ub (30). *In vivo* studies have reported that weak interactions between the Ub-binding domain and monoubiquitin chains are passively regulated by various mechanisms such as aggregation of Ub, modification of Ub and concatenation of domains (31). Given that the substrate proteins of USPs contain numerous cell homeostasis regulators, oncoproteins and tumour suppressor proteins, some USPs may serve as targets for the development of anti-tumour drugs.

**Figure 1:**
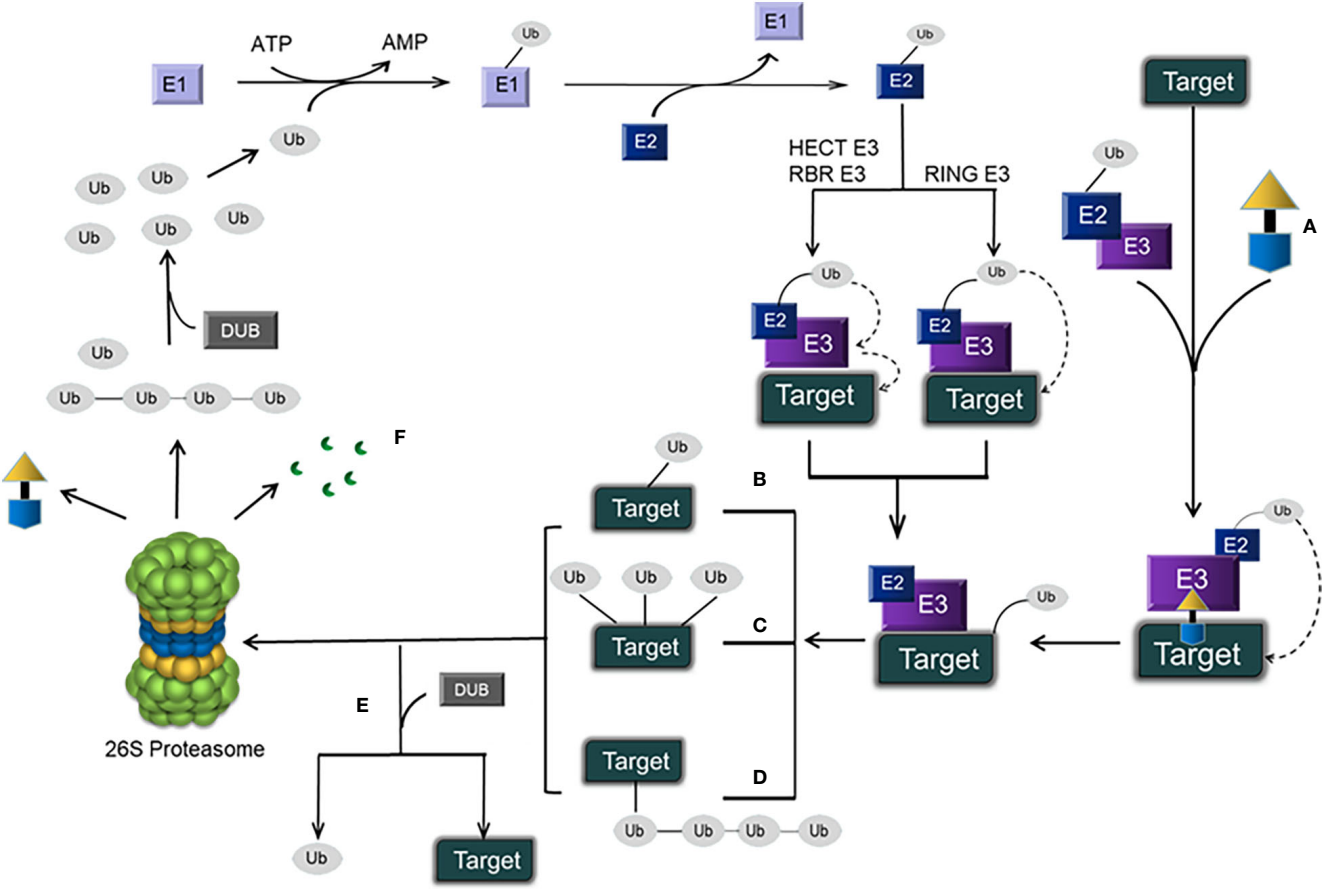
Schematic representation of the ubiquitin–proteasome pathway and PROTACs. **(A)** A PROTAC, which has an E3 ligase ligand at one end and a protein ligand at the other end, is connected by a linker in the middle to form a ternary complex: target protein–PROTAC–E3. Eventually, the target protein is degraded by the 26S proteasome, and the PROTAC is retained. **(B)** Monoubiquitination. **(C)** Multiubiquitination. **(D)** Polyubiquitination. **(E)** DUB-mediated deubiquitination (which disconnects target proteins from Ub). **(F)** Protein degradation products.

The ubiquitin-proteasome pathway has been shown in **Figure 1**.

## 3 PROTACs

Proteolysis-targeting chimaeras (PROTACs) are emerging small molecules that use UPP to degrade target proteins and have promoted unprecedented innovation in drug research and development. Multiple PROTACs are undergoing evaluation in phase I and II clinical trials (32–35). Advances in PROTAC technology may lead to the development of novel strategies for targeted therapy of cancer.

PROTAC is a chemical molecule containing different ligands at two ends. One end binds to E3 ligases, whereas the other end binds to intracellular proteins. The two ends are subsequently connected by a linker to form a ternary complex: target protein-PROTAC-E3 ligase. Such chemical molecules can be polyubiquitinated by recruiting targeted proteins to the vicinity of E3 ligases, resulting in the degradation of target proteins by the 26S proteasome. Owing to their recyclability, PROTACs are good candidates for cancer therapy (36, 37). For example, PROTAC ARV-110, which uses UPP to degrade the androgen receptor (AR) protein, has demonstrated some efficacy in the treatment of prostate cancer, especially metastatic castration-resistant prostate cancer (mCRPC). The mechanism of action of ARV-110 differs from that of traditional inhibitors. Because ARV-110 acts by degrading AR, instead of inhibiting AR function, it may help to overcome drug resistance (38).

PROTACs have several advantages over small-molecule inhibitors. First, PROTACs act by degrading target proteins instead of inhibiting their activity. Second, PROTACs may be used for selective targeting of target subtypes. They can inhibit the scaffold function of target proteins and improve the efficiency of the warhead. Third, because the protective effects of PROTACs can be achieved at lower doses, their use decreases the risk of dose-dependent toxicity and promotes long-term adherence (39). However, PROTACs have some disadvantages. Compared with the common small-molecule Ub ligase inhibitors, PROTACs have larger molecular weight and poor tissue and cell permeability in the human body. Moreover, their clinical safety as drugs warrants further investigation (40, 41).

The content of PROTACs has been shown in **Figure 1**.

## 4 Abnormally expressed USPs in tumour cells

To date, many studies have focused on evaluating the functions and substrates of proteins and the role of USPs in specific diseases. Mutations in USPs can disrupt cellular metabolism *in vivo*, resulting in an increased predisposition to cancer. Therefore, USPs can be used as targets for developing anticancer drugs (28). The common abnormal USPs associated with NSCLC and other tumours are summarised below.

### 4.1 USP4

The human USP4 gene was identified as a proto-oncogene related to Tre2/Tre17(USP6). In USP4, its catalytic domain is composed of a Cys box and His box, whereas its non-catalytic structure is composed of a USP domain (DUSP) and a Ub-like domain (UBL) (42). Studies have demonstrated that USP4 is closely associated with various malignant tumours, such as colorectal, breast, liver and lung cancers (43–46).

In breast cancer, USP4 directly interacts with and deubiquitinates TβRI and promotes the proliferation of tumor cells (44). In liver cancer, the low expression of microRNA-148a can increase the expression of the downstream molecule USP4, thereby promoting the proliferation and migration of liver cancer cells (45). In human osteosarcoma cells, USP4 can directly bind to and deubiquitinate ARF-BP1, which stabilises ARF-BP1 and reduces p53 levels, thereby promoting cancer cell proliferation (47).

USP4 may act as a tumour suppressor protein in some tumours. In head and neck squamous cell carcinoma, USP4 inhibits carcinogenesis by deubiquitinating receptor-interacting protein 1 (RIP1) and promoting apoptosis induced by tumour necrosis factor-α (TNF-α) (48). Downregulation of endogenous USP4 can promote TNF-α-induced migration of LUAD (A549) cells, indicating that USP4 is a negative regulator of cell migration. Mechanistically, USP4 can directly bind to the TRAF domain to inhibit TRAF2/TRAF6 activity through deubiquitination in an activity-dependent manner and negatively regulate IL-1β and TNF-α-induced NF-κB activation, thereby inhibiting lung cancer metastasis (46).

Therefore, USP4 plays different roles in different types of cancer cells, and whether it promotes or suppresses cancer remains controversial.

### 4.2 USP7

To date, USP7 is the most extensively investigated DUB and is the first DUB that can be specifically targeted and inhibited by drugs with promising therapeutic effects (49). It contains 1102 amino acid residues, has a molecular weight of approximately 135 kDa, and contains seven domains: an N-terminal TRAF-like (Tumor necrosis factor Receptor-Associated Factor) domain, a catalytic domain and five C-terminal ubiquitin-like domains (50, 51). MDM2 is one of the substrates of USP7. USP7 has a high affinity for MDM2, which often leads to p53 degradation and increases the risk of cancer. Therefore, USP7 can be considered an oncogene (52).

USP7 can deubiquitinate CCDC6, a tumour suppressor protein, in cells with DNA damage to protect it from degradation by the tumour suppressor FBXW7, thus improving the prognosis of NSCLC (53, 54).

Both USP7 and Ki-67 are highly expressed in NSCLC. In particular, Ki-67 expression is high in the G1, S, G2 and mitotic phases of the cell cycle, which usually indicates a poor prognosis. USP7 acts by binding to Ki-67 and stabilizing its expression to play a pro-carcinogenic role (55).

Fat mass and obesity associated protein (FTO), a demethylase that is significantly upregulated in NSCLC, can promote the expression of USP7 by activating its m6A demethylase activity, reducing the m6A level of USP7 and improving the stability of its mRNA (56).

In addition, USP7 is associated with phosphatase and tensin homologue (PTEN). In chronic myeloid leukaemia (CML), BCR-ABL can enhance USP7-induced deubiquitination of PTEN, which is conducive to nuclear rejection (57). *NPM1* is frequently mutated and can interact with USP7 in acute myeloid leukaemia (AML). It prevents USP7-mediated deubiquitination in the nucleus and promotes the translocation of PTEN to the cytoplasm, leading to polyubiquitination and degradation of PTEN in the cytoplasm (58). Patients with acute promyelocytic leukaemia (PML) harbour a PML-RARα translocation in which USP7 deubiquitinates PTEN primarily in the cytoplasm, promoting nuclear rejection (59).

### 4.3 USP10

The target proteins of USP10 include p53 (60), PTEN (61), P14ARF (62). Similar to that of USP7, the most prominent physiological function of USP10 is to specifically deubiquitinate p53 and neutralise the effects of MDM2, eventually stabilising p53 in normal cells (63). However, USP10 can stabilise oncogenes such as *FLT3* (64), *RAF1* (65) and *Musashi2* (66). USP10 overexpression in breast cancer and neuroblastoma is found to be associated with a poor prognosis (67, 68).

USP10 expression is downregulated in some cancers, including gastric cancer and NSCLC. Downregulation of USP10 results in a poor prognosis and shortened survival in gastric cancer. In NSCLC, USP10 deficiency is not significantly associated with clinical outcomes and prognosis, indicating that USP10 may not be directly involved in tumour progression (69, 70). However, USP10 can interact with PTEN to reduce K63-linked ubiquitination of PTEN mediated by the E3 ligase TRIM25, restore PTEN activity and reduce PIP3 production, thereby inhibiting the signal transduction of mammalian target of rapamycin (mTOR) in NSCLC cells (71). Although USP10 may act as a tumour suppressor, its role in different tumours remains controversial.

### 4.4 USP22

USP22 is highly expressed in various tumours such as lung (72), colorectal (73) and gastric cancers (74), and is involved in DNA transcription, malignant transformation of cells and cell cycle progression (75).

USP22 can be inhibited by shRNA, activates the p53 pathway in tumours and downregulates MDMX protein, thereby inducing apoptosis in NSCLC cells (9). In LUAD, knocking down USP22 may induce ubiquitin C (UBC) expression, which promotes cell cycle and ubiquitin-mediated protein degradation, and inhibits the occurrence of lysosomal autophagy, thereby promoting the development and progression of LUAD (76).

In addition, USP22 can promote hypoxia-induced generation of liver cancer stem cells through the HIF1α/USP22 positive feedback loop after p53 inactivation, and lipoprotein complexes targeting USP22 can inhibit the growth of liver cancer cells and enhance sorafenib sensitivity (77).

Therefore, USP22 may be a potential drug target for cancer therapy.

### 4.5 Other USPs

USP3 can interact with KLF5 and stabilize KLF5 through deubiquitination. USP3 knockdown inhibited the proliferation of breast cancer cells, while ectopic expression of KLF5 partially rescued this inhibitory effect (78). As one of the factors responsible for NSCLC cell proliferation, USP3 may lead to Ub-mediated degradation by targeting *RBM4*, which is a tumour suppressor gene and a key molecule for RNA splicing (79).

USP5 can enhance the stability of cyclin D1 (CCND1) protein, significantly prolong the half-life of CCND1 and reduce the degradation of CCND1. Additionally, it can promote the proliferative, migratory and colony-forming abilities of NSCLC cells. The USP5 inhibitor G9 can lead to cell cycle arrest in NSCLC cells and can significantly downregulate phosphorylated retinoblastoma (RB) protein, thereby inhibiting the growth of NSCLC xenografts (80). USP5 can activate the Wnt/β-catenin signalling pathway, and its expression is higher in NSCLC tissues than in normal tissues. It promotes nuclear accumulation and signalling of β-catenin by deubiquitinating it, and its increased expression is associated with large tumour size, poor differentiation, advanced tumour stage and poor patient survival (81).

Overexpression of USP14 promotes tumour cell proliferation and is associated with the poor prognosis of NSCLC. The mRNA expression of USP14, accumulation of β-catenin protein and activation of the Wnt pathway are upregulated in NSCLC, resulting in shorter survival (82). Therefore, USP14 acts as a tumour promoter and may serve as a promising therapeutic target for NSCLC.

USP17 is highly expressed in NSCLC, and its expression in squamous cell carcinoma is significantly higher than that in adenocarcinoma. The high expression of USP17 in squamous cell carcinoma may be attributed to differences in transcriptional regulation or protein turnover (83).

Overexpression of USP19 increases the migratory and invasive abilities of breast cancer (MCF7) cells. These effects of USP19 are closely related to its catalytic activity and transmembrane domain. Knockdown of USP19 reduces tumour aggressiveness, suggesting that USP19 plays a key role in the migration of breast cancer cells (84).

Overexpression of USP21 is associated with the progression of pancreatic ductal adenocarcinoma (PDAC). Overexpression of USP21 can accelerate PDAC growth in mice and promote the progression of pancreatic intraepithelial neoplasia (PanIN) to PDAC in immortalised human pancreatic ductal cell models. However, the loss of USP21 impairs PDAC cell growth. Mechanistically, USP21 promotes cancer cell proliferation by deubiquitinating and stabilising the TCF/LEF transcription factor TCF7 (85).

USP28 is upregulated in NSCLC and is associated with a poor prognosis of NSCLC (86). The lower expression of miR-3940-5p in NSCLC tissues than in paired adjacent normal tissues usually indicates tumor growth and proliferation (87). miR-3940-5p can target CCND1 and USP28 to inhibit the growth of NSCLC cells, and overexpression of CCND1 and USP28 can attenuate miR-3940-5P-induced proliferation and apoptosis of NSCLC cells (88). Additionally, USP28 plays an important role in maintaining the protein expression of c-MYC, c-JUN and Δp63 in laryngeal squamous cell carcinoma (LSCC). Inhibition of USP28 can reduce the expression of these proteins in mice with LSCC and induce tumour cell death (89).

USP29 can promote the migration of gastric cancer cells by preventing Snail degradation, thereby maintaining high protein expression of Snail in cancer cells. Snail is a major inducer of EMT and metastasis, and its high expression is associated with poor survival and prognosis (90). In addition, USP29 overexpression induced by chemotherapy and oxidative stress treatment can promote chemotherapy resistance by hijacking the USP29/Snail1 axis, which is associated with enhanced cancer stem cell properties, poor prognosis and drug resistance in lung cancer (91).

USP43, an H2BK120 deubiquitinase, exerts strong inhibitory effects on the growth and metastasis of breast cancer *in vivo*. It is phosphorylated by AKT in the cytoplasm and subsequently binds to the 14-3-3β/ε heterodimer to be retained in the cytoplasm, resulting in a marked reduction in USP43 levels in the nucleus. Low levels of USP43 in the nucleus are associated with EGFR accumulation, AKT hyperactivation, higher histological grades and a poor prognosis (92).

A list of USPs upregulated/downregulated in NSCLC is provided in **Table 1**.

**Table 1:** Ubiquitin-specific proteases upregulated/downregulated in NSCLC.

Deubiquitinase	Upregulated/ downregulated	NSCLC-related Events	Implicated signaling	References
**USP5**	Upregulated	USP5 promotes the proliferation and migration of NSCLC cells by binding to CCND1, reducing polyubiquitin-mediated degradation of CCND1 and stabilising its protein levels. KRAS activates USP5 to stabilise nuclear Beclin 1, leading to instability of MDM2-mediated p53 protein.	USP5/CCND1 and USP5/Beclin 1	(80, 93)
**USP7**	Upregulated	USP7 overexpression inhibits ERK1/2 through deubiquitination of RAF1 and inhibits the development of lung adenocarcinoma independently of p53 protein.	RAS/RAF/MEK/ERK	(7)
**USP9X**	Upregulated	USP9X interacts with prostaglandin E synthase (PTGES) to prevent its ubiquitination and degradation, enhance PGE2 signalling and promote NSCLC.	PTGES/PGE2	(94)
**USP10**	Downregulated	Loss of USP10 downregulates KLF4 expression and accelerates *KRAS* ^G12D^-driven initiation and progression of lung adenocarcinoma.	USP10/KLF4/TIMP3	(95)
**USP21**	Upregulated	USP21 deubiquitinates and stabilises the oncogene *YinYang-1* (*YY1*) in NSCLC cells, activates SNHG16 and promotes the proliferation of NSCLC cells.	USP21/YY1/SNHG16	(8)
**USP22**	Upregulated	Silencing USP22 using shRNA can downregulate MDMX, activate p53 pathway and inhibit the proliferation of NSCLC cells.	MDMX/P53	(9)
**USP28**	Upregulated	USP28 interacts with STAT3 to reduce STAT3 ubiquitination, increase STAT3 stability and promote NSCLC proliferation.	USP28/STAT3	(6)
**USP29**	Upregulated	USP29 hijacks the USP29/Snail1 axis to promote chemotherapy resistance in NSCLC.	USP29/Snail1	(91)
**USP35**	Upregulated	USP35 binds to ferroportin (FPN) to reduce ferroptosis triggered by erastin/RSL3, thereby promoting NSCLC proliferation. Overexpression of USP35 stabilises RRBP1, inhibits endoplasmic reticulum stress-induced apoptosis and promotes NSCLC proliferation.	Ferroptosis pathway and USP35/RRBP1	(5, 96)
**USP51**	Upregulated	CDK4/6 mediates the phosphorylation of USP51 at Ser26. Phosphorylated USP51 regulates the stability of ZEB1 protein through deubiquitination and promotes the metastasis of lung adenocarcinoma.	CDK4/6/USP51/ZEB1	(97)
**USP53**	Downregulated	USP53 deubiquitinates FKBP51, which in turn dephosphorylates AKT1 and ultimately inhibits tumor growth in LUAD.	FKBP51/AKT1	(98)

## 5 NSCLC-related targets and treatment

Signalling pathways associated with the development of NSCLC should be identified and examined to understand the pathogenesis of NSCLC. The PI3K/AKT/mTOR (99), Ras/Raf/Mek/Erk (100), Wnt/β-catenin (101), NF-κB pathways (102), are closely related to the occurrence and development of NSCLC. Inhibiting key proteins involved in these pathways may represent an effective therapeutic strategy for NSCLC. Similarly, tumour suppressor genes are often aberrantly expressed in NSCLC. For example, downregulation of p53 and PTEN promotes cancer cell proliferation and indicates a poor prognosis (103). Specific inhibitors can be used to target and degrade oncoproteins through UPP and activate tumour suppressor proteins, which can effectively block downstream signal transduction and prevent the proliferation of cancer cells. In recent years, PROTACs have demonstrated anti-tumour effects in cellular or preclinical models of NSCLC. The USPs and PROTAC targets related to NSCLC-associated signalling pathways are summarised below.

### 5.1 Targeting key proteins in tumour-related signalling pathways and application of PROTACs

#### 5.1.1 EGFR

Overexpression of Epidermal growth factor receptor (*EGFR*) is associated with the poor prognosis of several cancers, including oesophageal, breast, head and neck squamous cell, and lung cancers (104–107). In Asian and non-Asian populations, *EGFR* mutations are observed in approximately 40% and 20% of patients with NSCLC, respectively (108). Therefore, *EGFR* is one of the key therapeutic targets for NSCLC. To date, several generations of epithelial growth factor receptor tyrosine kinase inhibitors (EGFR-TKIs) have been used for the treatment of NSCLC. EGFR-TKIs can inhibit the proliferation and metastasis of tumour cells by mimicking ATP configuration and occupying ATP-binding sites, thereby blocking downstream signal transduction (109, 110). However, owing to mutations and signal bypass pathways, resistance to EGFR-TKIs has become a common problem in clinical settings (111). In lung adenocarcinoma, USP22 can induce EGFR-TKI resistance by regulating the endocytosis and transport of EGFR through deubiquitination modification, stabilising the intracellular EGFR protein levels and promoting the continuous activation of EGFR-dependent signalling pathways (112). Similarly, tripartite motif 25 (TRIM25), an E3 ligase, can promote the proliferation of cancer cells by promoting the K63-linked ubiquitination of EGFR, increasing the stability of EGFR protein and promoting the continuous activation of downstream signalling pathways (113).

In recent years, PROTACs have demonstrated good EGFR-targeting ability. Yu et al. developed MS 9427, an EGFR-specific PROTAC degrader that effectively induces efficient degradation of mutant EGFR in a concentration- and time-dependent manner through UPP and the autophagy-lysosome pathway and exerts a strong inhibitory effect on NSCLC cell proliferation (114). Cheng et al. reported that the E3 ligases VHL and CRBN can recruit the EGFR degraders MS 39 and MS 154, respectively, to effectively induce the degradation of mutant EGFR in NSCLC in an E3 ligase-dependent manner (115). In addition, Zhao et al. reported that compound P3, which contains a purine structure that recruits VHL, can significantly induce degradation of mutant EGFR^del 19^, promote cell apoptosis, arrest the cell cycle and inhibit colony formation (116). The PROTACs SIAIS 125 and SIAIS 126 can selectively degrade EGFR^L858R+T790M^ resistance proteins in H1975 cells and exhibit potent and selective anti-tumour effects against EGFR-TKI-resistant lung cancer. Selective degradation of EGFR^Ex19del^ mutant protein in NSCLC (PC9) cells can induce apoptosis (117). Zhang et al. reported that the orally available PROTAC HJM-561 can overcome EGFR triple mutation-mediated resistance in NSCLC by specifically degrading mutant EGFR proteins. Additionally, HJM-561 can exhibit potent antitumour effects against EGFR^Del19/T790M/C797S^-driven Ba/F3 cell-derived xenograft (CDX) and patient-derived xenograft (PDX) models of NSCLC resistant to osimertinib (118). Overall, PROTACs targeting EGFR are excellent therapeutic candidates for overcoming EGFR-TKI-induced resistance.

#### 5.1.2 PI3K/AKT/mTOR

The PI3K/AKT/mTOR pathway plays a key role in tumour growth, invasion, metastasis, and angiogenesis (119). Activation of PI3K by upstream signalling molecules, such as EGFR or other receptor tyrosine kinases, can catalyse PIP3 production, thereby triggering the activation of the AKT/mTOR signalling cascade (120). AKT plays an important role in tumor growth and development, such as ovarian, breast and gastric cancers (121–123). K63-linked polyubiquitination of AKT at lysine 8 and 14, which is mediated by the E3 ligase TRAF6 or SKP2, can localise AKT to the cell membrane, thereby resulting in AKT activation and enhanced intracellular signalling (124).

USPs and E3 ligases are involved in the PI3K/AKT/mTOR pathway. As a tumour suppressor, USP53 can deubiquitinate FK506-binding protein 51 (FKBP51) to dephosphorylate AKT1 and inactivate downstream signalling pathways, thereby inhibiting the progression of LUAD (98).

Calcium- and integrin-binding protein 1 (*CIB1*) is an oncogene that regulates cell adhesion, migration and differentiation (125). The E3 ligase CHIP can activate K48-linked polyubiquitination, which targets CIB1 to inhibit AKT/mTOR signalling and EMT in LUAD (126). Although significant progress has been achieved in the development of inhibitors targeting specific proteins in the PI3K/AKT/mTOR pathway to treat NSCLC, acquired drug resistance remains inevitable. However, targeted degradation of AKT may help to overcome drug resistance in NSCLC, and may exert long-term pharmacological effects when compared with their inhibition (127, 128). PROTACs can control tumour growth and invasion by inducing the ubiquitination and degradation of AKT. In the first PROTAC targeting AKT, INY-03-041, GDC-0068 was used as the target head compound and lenalidomide was used as an E3 ligase ligand. It can effectively induce degradation of the three isoforms of AKT proteins (AKT1/2/3) (128). Similarly, MS 143, a potent AKT degrader, can effectively inhibit tumour growth in xenograft mouse models by hijacking UPP to induce rapid and stable AKT degradation in a concentration- and time-dependent manner (129). However, to date, PROTACs targeting AKT in NSCLC have not been developed and warrant further exploration.

#### 5.1.3 RAS/RAF/MEK/ERK

The RAS/RAF/MEK/ERK signalling pathway, one of the MAPK cascades in humans, plays a key role in regulating various processes, including cell proliferation, differentiation and apoptosis (130, 131). After EGFR is activated by TGF-α, RAS, one of the downstream signals of EGFR, is activated initially through SOS and Grb2 and subsequently through a cascade of RAF and MEK1/2, eventually resulting in activation of ERK1/2 (132).

Studies have shown that USP7 may be involved in the regulation of RAF1 in lung adenocarcinoma cells in this signaling pathway. Overexpression of USP7 can regulate ERK1/2 signaling pathway, increase P53 expression, and inhibit G2/M transformation and proliferation of LUAD cells by deubiquitinating the M1, K6, K11, K27, K33, and K48 polyubiquitin chains of Raf-1 to reduce the level of RAF1 phosphorylation (7). In chronic myeloid leukemia cells, Leucine zipper-like transcriptional regulator 1 (LZTR1) participates in the formation of the junction of Cullin 3 (CUL3) ubiquitin ligase complex, mediates CUL3 and thus causes RAS ubiquitination, And regulate downstream MEK, ERK and other signals, and finally achieve the purpose of affecting BCR-ABL TKI (133).

KRAS is the predominant mutant subtype of the RAS family, accounting for approximately 85% of the total RAS mutations. *KRAS* ^G12C^ mutation accounts for approximately half of *KRAS* mutations in patients with LUAD (134, 135). KRAS is difficult to target owing to its lack of a deep binding pocket, its high affinity for GTP and the presence of abundant intracellular levels of GTP (136, 137). PROTACs can be used to overcome KRAS^12C^-induced resistance and target other KRAS mutants. For example, a VHL-based PROTAC has been designed using MRTX849 as a covalent KRAS^G12C^ warhead. The lead molecule LC-2 in this PROTAC can induce KRAS degradation and impair downstream MAPK signalling, resulting in reduced pERK levels in *KRAS* ^G12C^ mutant-positive human lung cancer cell lines (138). Lu et al. developed a reversible covalent PROTAC that can efficiently target the degradation of endogenous *KRAS* ^G12C^ in *KRAS* ^G12C^-mutated H358 and H23 lung cancer cells (139).

Furthermore, PROTACs have demonstrated great potential in targeting MEK protein for tumour therapy. The PROTAC MS432 has been synthesised by linking the major portion of PD0325901, a non-ATP competitive MEK inhibitor, to VHL or CRBN E3 ligase. It can effectively degrade MEK1/2 protein and effectively inhibit the proliferation of *BRAF*-mutated colorectal cancer (HT-29) cells and melanoma (SK-MEL-28) cells (140).

#### 5.1.4 Wnt/β-catenin

Wnt signalling was first found to play a role in tumour and embryonic development. Recent studies have revealed that it also plays a role in tissue regeneration in adult bones, skin and intestines (141, 142). Wnt/β-catenin is one of the branches of the Wnt signalling pathway. The modification and degradation of β-catenin protein is the key function of this signalling pathway, which can be regulated through ubiquitination and deubiquitination. The interaction between β-catenin and E-cadherin may be associated with the prognosis of NSCLC (143–145).

FBXW2, an E3 ligase, binds to β-catenin *via* EGF-AKT1-mediated phosphorylation at Ser552 and promotes its ubiquitination and degradation. Functionally, FBXW2 overexpression inhibits the migratory and invasive abilities of lung cancer cells by blocking β-catenin driven trans-activation of MMPs, whereas FXBW2 knockdown promotes the migratory and invasive abilities of lung cancer cells and tumour metastasis *in vitro* and *in vivo* (146), indicating that FBXW2 acts as a tumour suppressor.

USP44 may serve as a tumour suppressor protein in colorectal cancer (CRC). USP44 is significantly downregulated in CRC and enhances the apoptosis of CRC cells. Its overexpression increases the expression of Axin1 protein, a scaffolding protein that promotes negative regulation of the Wnt signalling pathway; reduces the expression of β-catenin, c-myc and cyclin D1 and inhibits Wnt/β-catenin signalling-mediated activation of apoptosis in CRC cells (147).

As an activator of the Wnt signalling pathway, USP7 may serve as a specific therapeutic target for APC-mutated CRC. USP7 stabilizes β-catenin through deubiquitination and activates the Wnt signalling pathway, whereas the β-catenin inhibitory domain of APC protects β-catenin from deubiquitination by USP7 (148). P5091, a specific inhibitor of UPS7, inhibits the growth of CRC cells by promoting ubiquitination and degradation of β-catenin. Therefore, inhibition of USP7 can inhibit the activation of Wnt signalling by regulating the ubiquitination of β-catenin (149).

Chen and Hu et al. reported that the PROTAC xStAx-VHLL, which is designed to target the Wnt pathway, can trigger β-catenin degradation and effectively inhibit the survival of patient-derived CRC organoids through Wnt signalling blockade. Therefore, xStAx-VHLL can be used in the treatment of CRC (150).

#### 5.1.5 NF-κB

Nuclear factor-κb (NF-κB) is a family of transcription factors that play an important role in regulating immune response and inflammation. NF-κB regulates the expression of a variety of genes and plays a key role in the occurrence and development of tumors, such as proliferation, migration and apoptosis. Inhibition of NF-κB signaling can inhibit cancer progression (151). Ubiquitination/deubiquitination plays a key role in the activation of the NF-κB signalling pathway (152).

USP20 plays an important role in TNFα-induced NF-κB signalling by stabilising p62. Knockdown of USP20 and p62 in HeLa cells decreases cell viability and number while increasing the expression of cleaved caspase-8, caspase-3 and PARP (153).

USP4 can inhibit NF-κB activation through deubiquitination of TRAF2 and TRAF6, thereby promoting cell migration and invasion in lung cancer (46). Overexpression of wild-type USP4 in Hela cells can inhibit TAK1 polyubiquitination and NF-κB activation, whereas its knockdown can enhance polyubiquitination of TAK1 and phosphorylated IκBα and negatively regulate NF-κB activation induced by IL-1β, LPS and TGFβ (154).

USP18 interacts with the TAK1-TAB1 and IKKα/β-Nemo complexes to cleave the K63-linked polyubiquitin chain attached to TAK1 and inhibit NEMO ubiquitination, respectively, in a protein-dependent manner, thereby negatively regulating NF-κB (155).

#### 5.1.6 PTEN

PTEN is a tumour inhibitor that can antagonise PI3K function by dephosphorylating PIP3 to PIP2, thereby inactivating downstream oncogenic signalling and exerting tumour-suppressing effects (156, 157). PTEN inhibits cell proliferation and survival by inducing cell cycle arrest through the PI3K/AKT pathway or by downregulating cyclin D1 and decreasing its accumulation in the nucleus (158). However, owing to gene mutations, deletions or promoter methylation, PTEN expression is significantly reduced in NSCLC, which promotes tumour growth and invasion (159). Post-translational modifications (PTMS) of PTEN, including ubiquitination of PTEN, may represent an effective strategy for regulating PTEN function and serve as therapeutic targets for cancer (160).

Casein kinase 1-alpha (CK1α) competitively antagonises PTEN ubiquitination mediated by the E3 ligase NEDD4-1 by binding to the C-terminus of PTEN. Additionally, it positively regulates autophagy and inhibits the growth of NSCLC cells (161). Similarly, the deubiquitylase OTUD3 can interact with PTEN and deubiquitinate it, thus stabilising PTEN and inhibiting the development of breast cancer (162). However, in lung cancer, OTUD3 promotes tumour proliferation and metastasis and inhibits apoptosis through deubiquitination of GRP78 protein and is closely related to drug resistance (163). Therefore, most deubiquitinating enzymes play different roles in different tumours, which should be comprehensively investigated for the precise treatment of tumours.

#### 5.1.7 MDM2/p53

p53 is a typical intracellular tumour suppressor that is activated in response to DNA damage or oncogene activation and can act as a negative regulator to induce cell cycle arrest, apoptosis and senescence in cancer cells, thereby inhibiting growth and proliferation. MDM2 suppresses p53 primarily by inhibiting its transcriptional activity and inducing its nuclear export and Ub-mediated degradation, thereby decreasing the p53 protein level to maintain normal cell function (164). Ubiquitination of p53 is a key regulatory event in the p53 pathway and can be reversed by DUBs (165). Therefore, reactivation of p53 in cancer cells by blocking the MDM2/p53 pathway is an effective approach for targeted therapy of cancer.

Several studies have demonstrated that USPs can regulate MDM2/p53. Abraxas brother 1 (ABRO1), a component of the BRCC36-containing isopeptidase complex (BRISC), stabilises p53 by promoting its interaction with USP7 to reduce p53 ubiquitination (166). USP15 knockdown can delay Ub-mediated degradation of p53 by accelerating MDM2 degradation in melanoma (A375) cells (167). USP2a regulates the p53 pathway by stabilising MDM2 and MdmX to promote the growth of testicular embryonal carcinoma (NTERA-2) cells and breast cancer (MCF7) cells (168).

Compound 11a, a PROTAC that induces MDM2 degradation, can induce proteasome-dependent degradation of MDM2 in NSCLC (A549) cells and effectively inhibit tumour growth in xenograft mouse models of LUAD (169).

#### 5.1.8 ALK

*ALK* rearrangement genes are detected in 3%–7% of NSCLC cases, with *EML4-ALK* being the most important fusion genes (170). ALK TKIs are the first-line treatment agents in patients with lung cancer who harbour *ALK* rearrangements. To date, six small-molecule inhibitors of ALK have been used to treat ALK-positive NSCLC: crizotinib, ceritinib, alectinib, brigatinib, ensartinib and lorlatinib (171). Although these inhibitors have demonstrated promising efficacy in clinical settings, serious challenges such as drug resistance and brain metastasis may emerge with their long-term use (172, 173).

PROTACs have unique advantages in targeting ALK. Gray and Jin et al. reported two sets of PROTACs: TL13-12 and TL13-112 and MS4077 and MS4078, which can mediate ubiquitination and degradation of NPM-ALK and EML4-ALK *in vitro* (174, 175). Xu et al. developed PROTACs compounds (36 and 37) with a novel skeleton based on the second-generation small-molecule inhibitor alectinib. These PROTACs induced strong anti-proliferative activity in H3122 (NSCLC cells expressing the EML4-ALK fusion protein) and Karpas 299 (anaplastic large cell lymphoma cells expressing the NPM-ALK fusion protein) cells without causing toxicity in A549 and HFL-1 cells (cell without ALK expression). These results indicated that the PROTACs had good selectivity (176).

Therefore, in addition to traditional chemical inhibitors, PROTACs are promising therapeutic agents for NSCLC and can delay the emergence of resistance mutations, thereby improving patient outcomes.

#### 5.1.9 The roles of PROTACs in other targets associated with NSCLC

Research into the use of PROTACs for targeting NSCLC-associated genes is gradually emerging.

*SHP2* is frequently activated in lung cancer (177). The SHP2-targeting PROTAC SHP2-D26 can induce rapid and efficient degradation of SHP2 and is 10- to 100-fold more effective than allosteric SHP inhibitors in inhibiting ERK activity in cancer cells (178).

Nuclear focal adhesion kinase (*FAK*) is overexpressed in various tumours, including lung cancer (179). The Fak-targeted PROTAC GSK215 can significantly inhibit the migration of A549 cells, and induce rapid and long-term degradation of FAK, with lasting effects on FAK levels (180).

The PROTAC MD13, which targets macrophage migration inhibitory factor (MIF), can effectively inhibit the proliferation of A549 cells, resulting in cell cycle arrest at the G2/M phase (181).

Bromodomain-containing protein 4 (BRD4) is abnormally upregulated in lung cancer (182). The BRD4-targeting PROTAC CREATE can reshape the tumour microenvironment to directly reduce TAMs and effectively treat lung cancer (183).

In conclusion, PROTACs represent a novel therapeutic modality for NSCLC and other cancers.

### 5.2 Application of USP-related small-molecule inhibitors in cancer

Various partial and specific inhibitors of USPs have been developed for the treatment of cancer. Some USP inhibitors used in the treatment of NSCLC are shown in **Table 2**.

**Table 2:** Specific protease inhibitors used in the treatment of NSCLC.

USPs	Inhibitor	Mechanisms of inhibition	Signalling pathway	References
**USP1**	pimozide or GW7647	It can affect the formation of the Fanconi anaemia core complex and reverse the resistance of NSCLC cells to the DNA cross-linking agent cisplatin.	FA/BRCA	(184, 185)
**USP5**	G9	G9 can significantly reduce the expression of CCND1 protein in NSCLC tissues and inhibit the progression of NSCLC.	USP5/CCND1	(80)
**USP7**	P5091 or P22027	The inhibition of FTO promoted the m6A methylation level of USP7 mRNA and reduced the stability of USP7 mRNA, thereby inhibiting NSCLC.	FTO/USP7/MDM2/P53	(56)
**USP9X**	WP1130	Downregulation of USP9X increases the sensitivity of NSCLC cells to cisplatin by inhibiting p53.	USP9X/P53	(186)
**USP14**	IU1-47 or siRNA-USP14	It can arrest A549 cells at the G2/M phase.	IRE1/XBP1, JNK1 and PERK/eIF2α	(187, 188)
**USP28**	FT206	It can decrease the expression of c- MYC, c- JUN, and Δp63 and induce the regression of lung squamous cell carcinoma.	USP28/c-MYC, USP28/c-JUN and USP28/Δp63	(89)

LCA hydroxyamide (LCAHA) is a USP2a inhibitor that induces G0/G1-phase arrest by inhibiting the deubiquitinase USP2a and destabilising cyclin D1 and is independent of p53 status (189). FT671, a noncovalent inhibitor of USP7, can increase the degradation of the Ub ligase MDM2 and the expression of p53 to exert tumour-suppressing effects *in vivo* (190). In addition, USP7 inhibitor P22077 can effectively induce apoptosis of cancer cells in neuroblastoma cells with intact USP7/HDM2/p53 axis (191). WP1130 is an inhibitor of DUBs that inhibits activities such as USP5, UCH-L1, USP9X, USP14, and UCH37 (192). Combined treatment with WP1130 and cisplatin can synergistically inhibit the viability of LUAD (A549 and HCC827) cells (186). EOAI3402143(G9) is a non-specific USP inhibitor that inhibits USP5, USP9X and USP24 in a dose-dependent manner. Compared with WP1130, G9 has greater solubility and inhibitory activity against USP5 and USP9X (80, 193, 194), and the therapeutic role of G9 has been investigated in human pancreatic cancer (195). GSK2643943A, an inhibitor of USP20, has been investigated in oral squamous cell carcinoma (OSCC) (196).

### 5.3 Targeting of E3 ligases

Proteasome inhibitors, such as bortezomib, have demonstrated good efficacy in the treatment of multiple myeloma, which indicates the feasibility of research and development of drugs targeting UPP. However, because these inhibitors inhibit all 26S proteasome-dependent protein degradation pathways without discrimination, they can cause damage to normal cells and have significant side effects (197). Among the potential drug targets of ubiquitin-protease system, E3 ligases have outstanding advantages: the specific structure of some E3 ligases determines that they have better selectivity for screening small molecule inhibitors or developing specific targeted antibodies.

The E3 ligase CRBN is the target of many drugs, and several multi-target PROTACs based on CRBN have been developed (198). For example, ARV-825, a CRBN-based anti-BET PROTAC, exploits the association between CRBN and OTX015 to promote BRD4 degradation and inhibit cancer cell progression (199). Lenalidomide exerts significant activity by inhibiting CRBN, which forms a new substrate bound by the cullin-RING ligase 4 (CRL4) complex, leading to ubiquitination and proteasome-dependent degradation, resulting in antimyeloma activity (200).

The E3 ligase Skp2 is overexpressed in many human cancers and can regulate tumorigenesis (201). The Skp2 inhibitor SMIP004 can improve the efficacy of radiotherapy; upregulate programmed cell death protein 4 (PDCD4) expression levels, the target of Skp2; and inhibit the proliferation of breast cancer cells (202).

Compound A1874 synthesised using mouse double minute 2 (MDM2) as an E3 ligase receptor can degrade bromodomain-containing protein 4 (BRD4), upregulate the tumour suppressor p53, activate caspase-related apoptosis of colon cancer cells and effectively inhibit the viability of colon cancer cells (203, 204).

AMG-232, a small-molecule inhibitor of piperidinones that targets the E3 ligase, induces p53 activity, thereby arresting the cell cycle and inhibiting tumour cell proliferation in melanoma (205, 206).

The PROTAC ARV-471 is a protein degrader that binds to E3 ligases and oestrogen receptor (ER) and has potential applications in the treatment of breast cancer (207).

In conclusion, targeting E3 ligases represents an effective strategy for the treatment of NSCLC.

## 6 Concluding remarks

In this review, we summarised the role of USPs and E3 ligases in UPP and NSCLC-associated signalling pathways and discussed the research progress of corresponding targeted drugs, PROTACs and small-molecule inhibitors. Significant advancements have been made in the field of USPs; however, the role of USPs and E3 ligase-mediated PROTACs in the signal transduction of target protein degradation warrants further investigation. USPs and E3 ligases are aberrantly expressed in many tumours and can be used to regulate the activity of cancer cells. To date, studies have majorly focused on the following aspects: identifying oncogenic proteins or enzymes and understanding their pathogenic mechanisms and targeting and inducing ubiquitination and degradation of related substrates for the treatment of cancer. Given that promoting the degradation of USPs and enhancing the activity of E3 ligases are beneficial approaches for cancer therapy, the high expression of USPs in cells can be inhibited to shift the intracellular balance to E3 ligases that promote the degradation of target proteins. However, this viewpoint is complicated because target proteins in different cancers play different roles, and the corresponding specific USPs or E3 ligases also act as tumour promoters and/or suppressors. In addition, most related studies are based on carcinogenic USPs, whereas studies on tumour-suppressing USPs are limited. Moreover, the mechanisms underlying USP-induced drug resistance in tumours remain unclear. USPs are involved in not only cancer but also other diseases, such as neurodegenerative diseases, immune diseases, alcohol-associated liver disease and chronic kidney disease (208–211). When considering the function of USPs as an oncogene or tumor inhibitor, this knowledge needs to be combined with a variety of unknown substrate proteins and tissue and/or cell-specific aspects that will improve our understanding of the pathogenesis of NSCLC and may contribute to the development of future therapies.

## Author contributions

JZ and L-TM participated in supervision, conception, and editing. Y-CY, C-JZ and Z-FJ participated in writing the manuscript, writing-review. All authors contributed to the article and approved the submitted version.
